# Tilting deformation analysis and instability prediction of arch-locked-segment landslides induced by rainfall

**DOI:** 10.1038/s41598-025-88186-y

**Published:** 2025-01-27

**Authors:** Jingjing Liu, Handong Liu, Jiaming Luo, Jiaxing Chen, Hu Wang

**Affiliations:** 1 College of Geosciences and Engineering, North China University of Water Resources and Electric Power, Zhengzhou, 450046 China; 2 Henan Key Laboratory of Geomechanics and Structure Engineering, Zhengzhou, 450045 China

**Keywords:** Arch-locked-segment landslide, Model testing, Tilting deformation, Evolution process, Landslide prediction, Natural hazards, Environmental impact

## Abstract

The failure of locked-segment landslides is associated with the destruction of locked segments that exhibit an energy accumulation effect. Thus, understanding their failure mode and instability mechanism for landslide hazard prevention and control is critical. In this paper, multiple instruments, such as tilt sensors, pore water pressure gauges, moisture sensors, matrix suction sensors, resistance strain gauges, miniature earth pressure sensors, a three-dimensional (3D) laser scanner, and a camera, were used to conduct the physical model tests on the rainfall-induced arch locked-segment landslide to analyze the resulting tilting deformation and evolution mechanism. The results indicate that the tilting deformation characteristics in the locked segment are consistent with the variation in its strain, stress, hydrodynamic responses, and slope morphology, suggesting that tilting deformation can serve as a novel monitoring approach for landslide instability. Further, the tangent angle method and the tilting rate reciprocal method can be utilized to predict the landslide instability based on the landslide tilting deformation curve. The effectiveness of this method is validated in the Huangzangsi dam area, which provides theoretical foundations for understanding the catastrophic mechanism and instability prediction of arch-locked-segment landslides.

## 1. Introduction

Landslide disasters are widespread all over the world, especially in areas with complex terrain, active geological structure and changeable climate. The unstable geological conditions in these areas are easily affected by natural factors such as earthquakes and rainfall, resulting in frequent landslides and posing a serious threat to human life and property safety^1^. Through in-depth analysis of the cause mechanism, evolution process and influencing factors of landslide disasters, relevant scholars have gradually revealed the complexity of landslide^2–7^, and have established the concept of “locked-segment landslide” as a typical type of landslide hazard^8–11^. It has been shown that locked-segment landslides exhibit the energy accumulation effect. The stress on the locked segment exceeding its long-term strength suddenly releases a large amount of strain energy accumulated inside it, causing brittle failure to the locked segment, which initiates high-speed remote movement for the sliding mass and leads to a high-speed remote landslide. It is found that landslides with different locked segment types have distinct instability mechanisms^12–14^.

Based on extensive studies on geological conditions, triggering factors, instability patterns, and evolution mechanisms of landslide hazards have been identified. Huang et al.^15,16^ classified locked-segment landslides into three-segment type, retaining wall type, block resistance type, linear multi-level type, and stepped multi-level type, and systematically summarized factors initiating instability of various locked-segment landslides based on their dynamic evolution characteristics. Based on the locked segment type and geological conditions, Pan et al.^14^ classified locked-segment landslides into cross-layer oblique shear type, parallel-layer direct shear type, homogeneous rock bridge type, retaining wall type, and supporting arch type. By studying geological conditions and the triggering factors for landslides in western Henan Province, Liu et al.^17,18^ identified that the main types of locked-segment landslides in this region are parallel-layer direct shear type, cross-layer oblique shear type, and retaining wall type. Large-scale physical model tests were conducted to ascertain the criteria for the evolution states of locked-segment landslides in western Henan Province and reveal the corresponding catastrophic mechanism. The locked segment is crucial in the deformation and stability of landslides. Thus, studying the failure modes and evolution mechanism of the locked segment is imperative in understanding and predicting landslide hazards.

Arch-locked-segment landslide is a particular type of landslide characterized by the sliding mass constrained by the terrain on both sides, forming a shape similar to an arch. Currently, there is little research on arch-locked-segment landslides. Dong et al.^19^ studied the locking effect at the arch section of the Maijianwo landslide through numerical analysis and highlighted issues pertaining to the failure modes of the locked segment and resulting landslides. Further, Liu et al.^20^ experimentally studied the physical model of the arch landslide and analyzed its instability mechanism. Jiang et al.^21^ investigated the stress-strain data characteristics and deformation patterns of arch-locked-segment landslides. These studies have offered insights into understanding and predicting the evolution mechanism of arch-locked-segment landslides and provided a scientific basis for further research on this type of landslide.

China is one of the countries most severely affected by landslide hazards globally. Accurately predicting landslides is direly needed for effective hazard prevention and mitigation. Over the past few decades, technological advancements have gradually replaced traditional point-based manual monitoring with high-precision, automated, and distributed real-time monitoring methods. Typical landslide early warning systems are based on slope displacement monitoring^22–24^. The installation and maintenance of most traditional sensors (including displacement meters) are complex, which increases costs and limits their applicability. Therefore, selecting appropriate and effective monitoring indicators for enhancing the reliability of landslide prediction is critical in hazard reduction and prevention. At present, the advanced techniques and methods of landslide research are being widely used, such as optical fiber sensing technology, neural network analysis, big data processing and machine learning algorithms. Fiber optic sensor is widely used in landslide deformation monitoring because of its high precision and strong anti-electromagnetic interference ability, and provides reliable data support for landslide early warning^25–28^. Through intelligent analysis and prediction of landslide monitoring data, neural network technology can effectively identify its deformation trend and potential risks^29–31^. The combination of big data processing and machine learning algorithms has further improved the accuracy and timeliness of landslide warning. The comprehensive application of these technologies provides a scientific basis for the prevention and reduction of landslide disasters, and improves the ability of landslide monitoring and early warning. In addition, Tilt sensors have high measurement accuracy, low cost, simple installation and maintenance, and real-time monitoring capabilities in landslide monitoring. Like landslide displacement monitoring, variation in tilting angle during landslide evolution is a more reliable and practical monitoring indicator. Liu et al.^32,33^ demonstrated the effectiveness of tilt sensors in landslide monitoring based on landslide tilting deformation model tests and based on the velocity reciprocal method and proposed a new landslide prediction method for predicting the locked-segment landslides. By utilizing the monitoring data from the tilt sensors, Uchimura et al.^34,35^ suggested that prevention should be issued at a tilting rate change of 0.01°/h, and early warning should be issued at a tilting rate change of 0.1°/h. Xie et al.^36,37^ proposed a linear relationship between the shallow landslide displacement and the tilting angle and developed a corresponding linear equation for predicting landslide instability. Through comparative studies, Wang^38^ showed that warning methods based on tilting angle variation are more practical and effective than those based on tilting angle, displacement, and other prediction parameters. The aforementioned studies indicate that the tilting deformation curve of the landslide effectively reflects its evolution process and is a critical parameter for landslide warning and prediction.

Physical model testing is one of the important methods for studying landslide deformation and failure and revealing its catastrophic mechanisms. By simulating geological conditions and triggering factors such as rainfall, reservoir water, and earthquakes, it is possible to qualitatively or quantitatively reflect the stress characteristics of landslide soil and rock mass, analyze the damage evolution and failure characteristics, and further reveal their mechanical mechanisms. In this paper, physical model tests were conducted on arch locked-segment landslides with inclination angle sensors as the primary monitoring equipment, supplemented by pore water pressure meters, moisture content sensors, matric suction sensors, resistive strain gauges, miniature soil pressure meters, 3D laser scanners, and cameras. The tilting deformation characteristics, locked-segment failure features, and landslide evolution mechanisms of arch locked-segment landslides under rainfall conditions were evaluated, and corresponding predictions for landslide instability were proposed.

## Testing apparatus and methods

### Test materials

The rainfall-induced arch-locked-segment landslide model testing system primarily consists of a landslide model, a rainfall control system, and a data monitoring system. The landslide model was 1.8 m in length, 0.6 m in width, and 0.6 m in height, comprising the bedrock, sliding mass, and locked segment. The bedrock was constructed from masonry blocks and mortar. The sliding mass was primarily silty clay, and its physical and mechanical parameters were determined by particle analysis, direct shear, compaction, and permeability tests (Table 1).

**Table 1:** Physical and mechanical parameters of the soil used for model test.

Material	Dry density /(g/cm^3^)	Water content/%	Cohesion /(kPa)	Internal friction angle/(°)	Permeability coefficient /(cm/s)
Sliding mass	1.52	16	8	31	3.5 × 10^− 5^

The arch-locked segment in the test was 0.15 m long, 0.24 m high, and 0.02 m thick. Gypsum mortar can be used to simulate the mechanical properties, i.e., strength, elastic modulus, and Poisson’s ratio, of the locked segment^17,39^. Based on a series of test analyses, a mixture of water, gypsum, and sand at a mass ratio 1:1.4:1 was determined to simulate the locked segment. Cylindrical samples were prepared using the same mix proportion, yielding an unconfined compressive strength of 3.27 MPa. A layer of paraffin wax was applied to the surface of the locked segment to ensure that its strength was not affected by rainfall infiltration.

### Testing apparatus

**Fig. 1:**
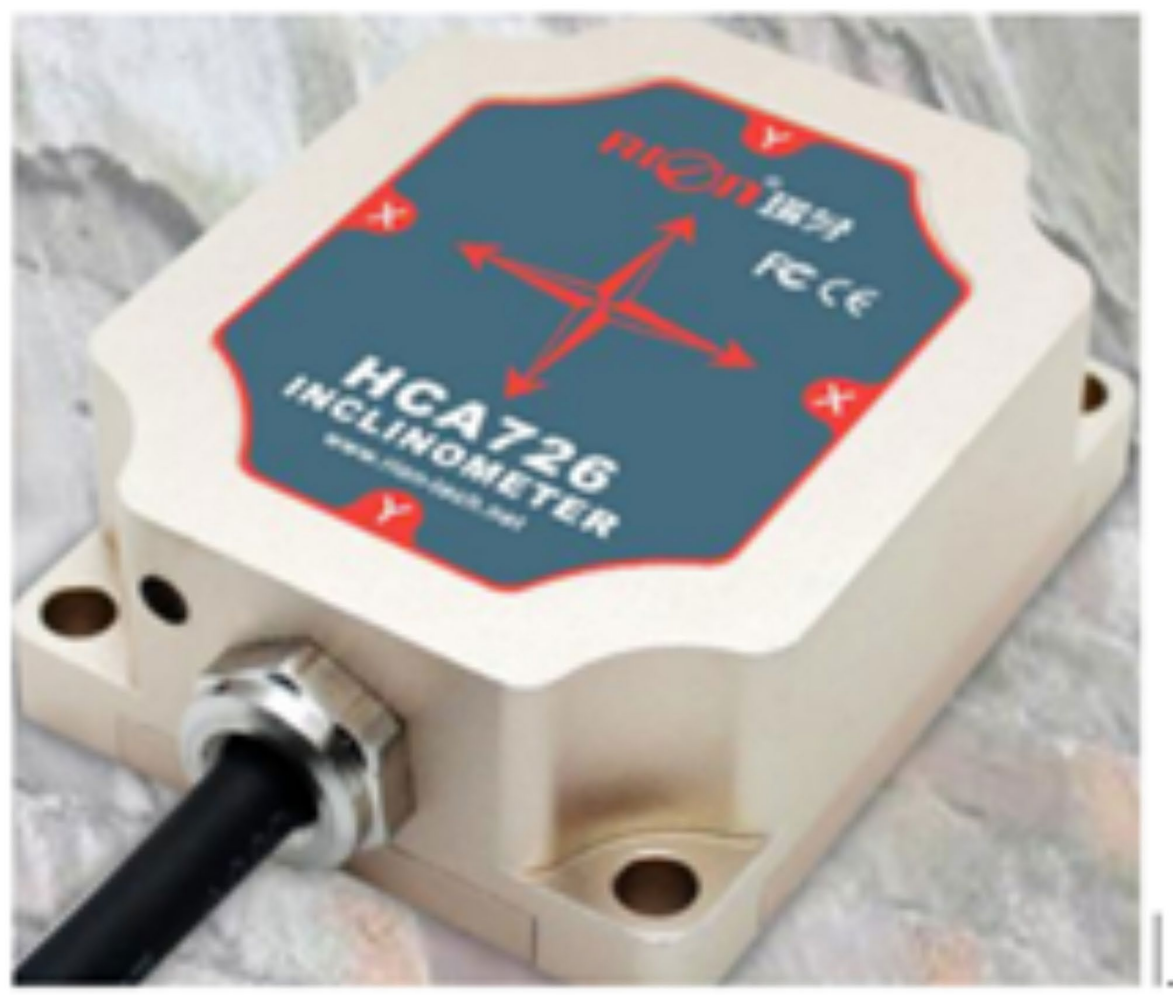
Dimensions of HCA726 tilt sensor.

**Fig. 2:**
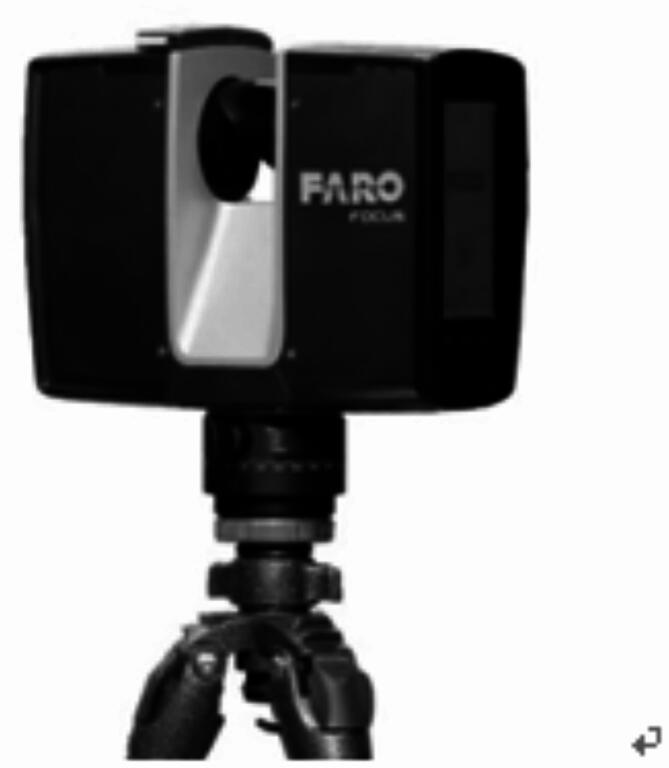
FARO X330 3D laser scanner.

The rainfall apparatus was a portable artificial rainfall system. The monitoring equipment included low-cost, high-precision instruments such as HCA726S dual-axis tilt sensors, DMTY pore water pressure sensors, Meter EC-5 moisture sensors, TEROS 21 matrix suction sensors, strain gauges, DMTY miniature earth pressure sensors, a 3D laser scanner, and a high-definition camera. The HCA726S tilt sensor (Fig. 1) incorporates advanced MEMS technology and can monitor the tilting deformation during landslide evolution. It measures 56 mm × 46 mm × 20.5 mm, with a range of ± 10°, an accuracy of ± 0.008°, a resolution of 0.0001°, and a data acquisition frequency of 15 Hz. It allows for dual-axis tilting angle measurement. The FARO X330 3D laser scanner (Fig. 2) has a horizontal scanning range of 360° and a vertical scanning range of 270°, with a scanning precision of ± 1 mm. This scanner captures the slope morphology at different times in the form of 3D point clouds, which can be utilized to construct 3D digital terrain models in Surfer software.

### Testing protocol

**Fig. 3:**
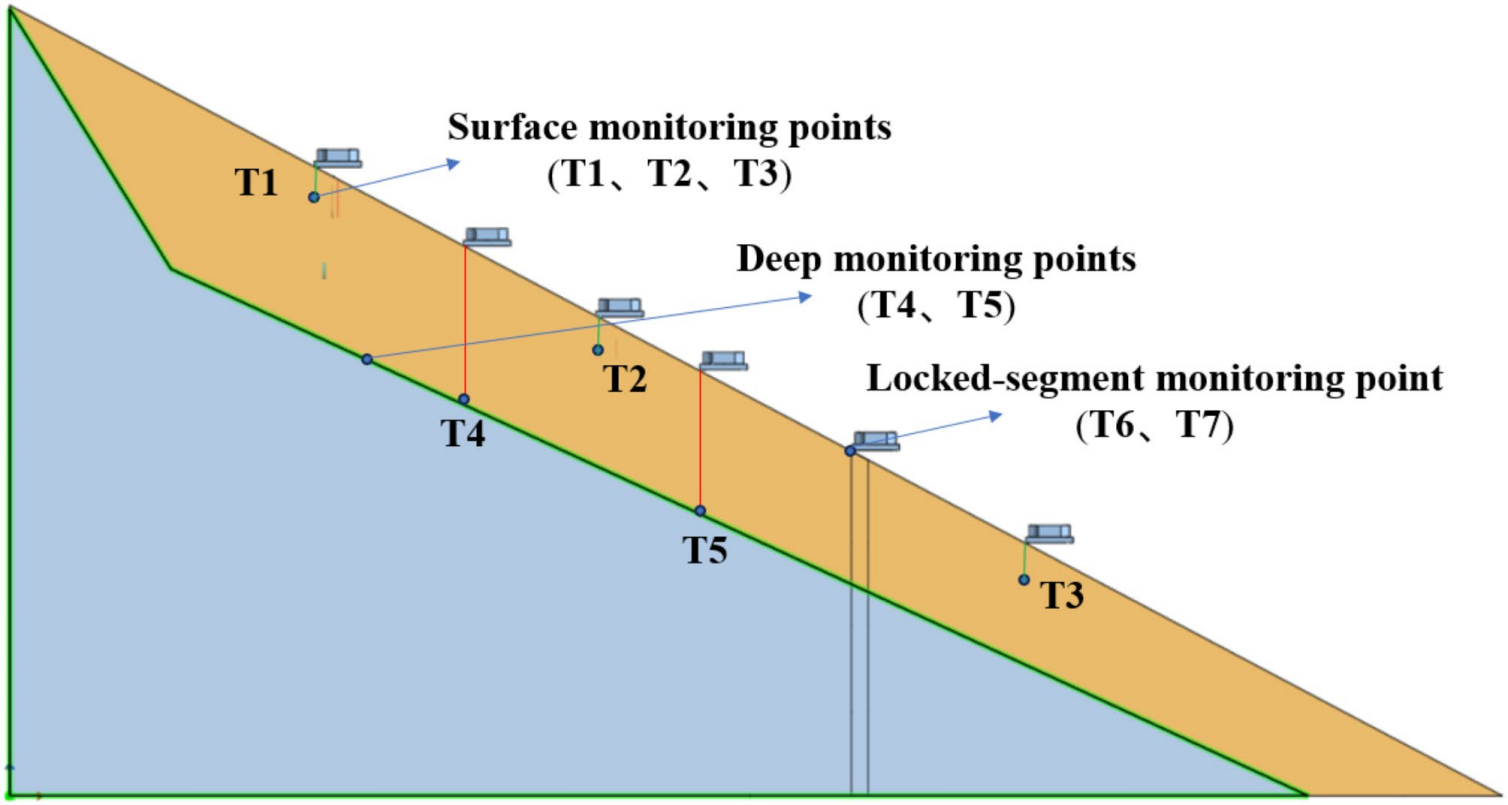
Layout of tilt sensor monitoring points.

**Fig. 4:**
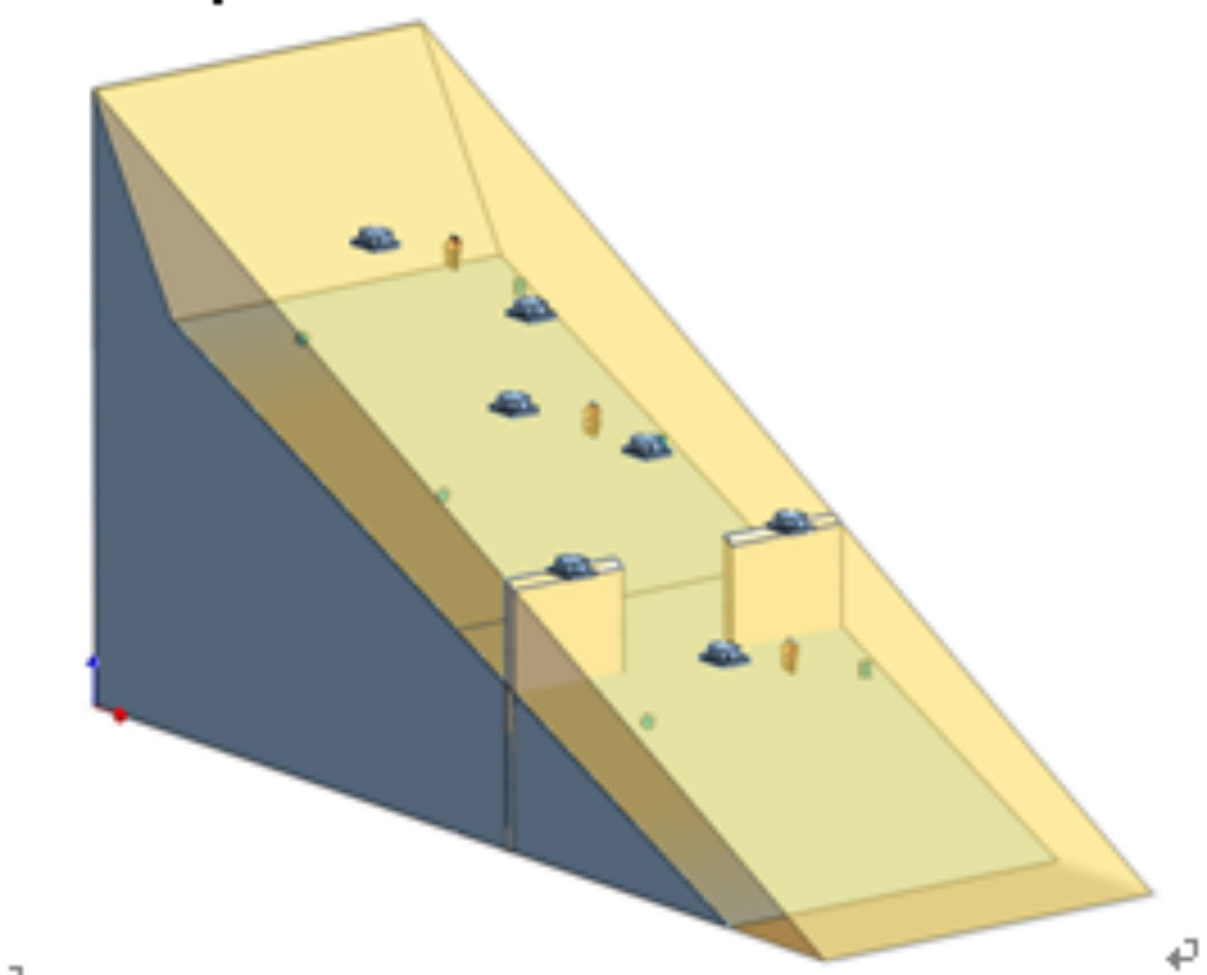
Arch-locked-segment landslide model.

Figure 3 shows the layout of the tilt sensor monitoring points. Tilt sensors T1 to T3 were installed on the upper, middle, and toe parts of the slope, respectively, at a distance of 6 cm from the slope surface to monitor the tilting deformation of the slope surface. Tilt sensors T4 and T5 were fixed to steel rods and embedded at the contact surface between the bedrock and sliding mass to monitor the deep deformation of the landslide. Tilt sensors T6 and T7 were fixed on the arch-locked segment to monitor its failure process. Pore water pressure sensors P1, P2, and P3, along with miniature earth pressure sensors S1, S2, and S3, were embedded 10 cm deep into the upper, middle, and lower parts of the slope surface to monitor the slope stress during landslide evolution. Sensors S4 and S5 were fixed on the side of the locked segment near the rear edge to monitor the stress changes experienced by the locked segment due to the slope behind it. Resistance strain gauges were installed at the contact interface between the locked segment and bedrock, which were protected for waterproofing. The strain gauges used are model BF120-20AA-X30, with a sensitivity of 2.0%, a resistance of 120.0 Ω, and a dynamic data acquisition frequency of 500 Hz. Figure 4 presents the physical model of the arch-locked-segment landslide. During the test, the 3D laser scanner was used to scan the model every 10 min, while the high-definition camera captured images of the slope every 5 min. Continuous video recording of the slope side was conducted to study the dynamic evolution process of the landslide, with a rainfall intensity set at 50 mm/h.

## Results and analysis

### Slope morphology and displacement characteristics of arch-locked-segment landslide

**Fig. 5:**
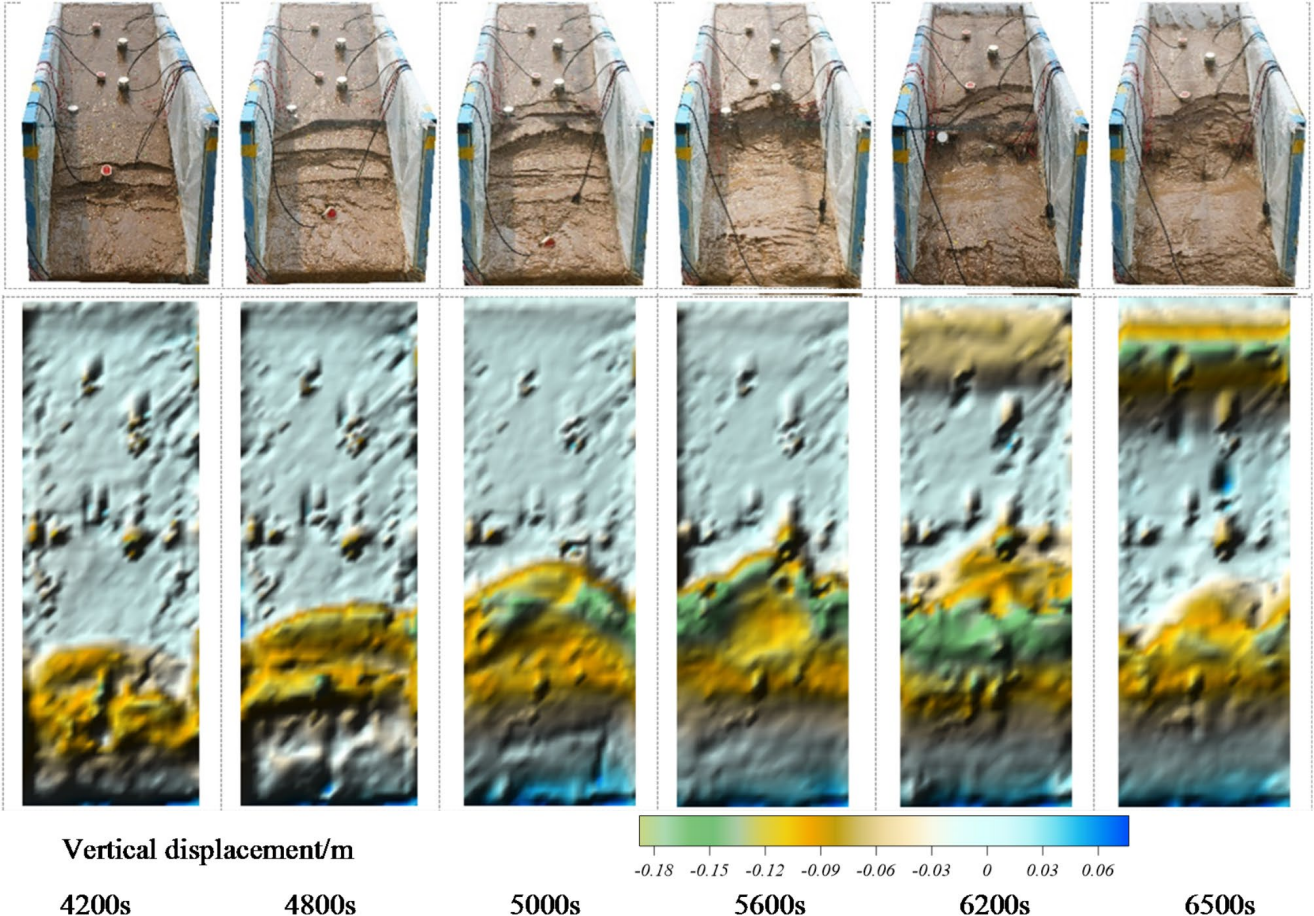
Slope morphology evolution and displacement nephograms of the arch-locked-segment landslide.

As shown in Fig. 5, the analysis of the evolution of the arch-locked-segment landslide under rainfall conditions reveals that the slope undergoes erosion at its slope toe first due to rainfall infiltration and surface runoff, leading to progressive damage as the rainfall continues. Around 4,200 s after the rainfall, the slope toe of the landslide experiences a stepwise upward collapse, causing tilt sensor T3 to fail. Around 4,800 s after the rainfall, the landslide develops progressively in a fan-shaped failure form to the landslide area near the locked segment influenced by the locked segment, with a vertical collapse displacement of about 0.05 m. At around 5,000 s, the failure extends from the slope toe to the middle of the landslide, forming a damaged arch structure in the locked segment with 0.1 m vertical collapse displacement. As rainfall continues, the landslide progresses upward. At around 5,600 s, the arch in the locked segment becomes prominent, and the locked segment transitions from a stable to an unstable state under the influence of rainfall. The rear edge of the landslide reaches saturation and begins to slide downward. Meanwhile, the rear thrust on the locked segment increases. At about 6,200 s, the arch-locked segment fractures and collapses, and overall instability and sliding of the landslide occur, with a rear edge settlement of about 0.05 m. After 300 s, the landslide rapidly collapses due to support loss from the segment.

### Response of hydrodynamic parameters

**Fig. 6:**
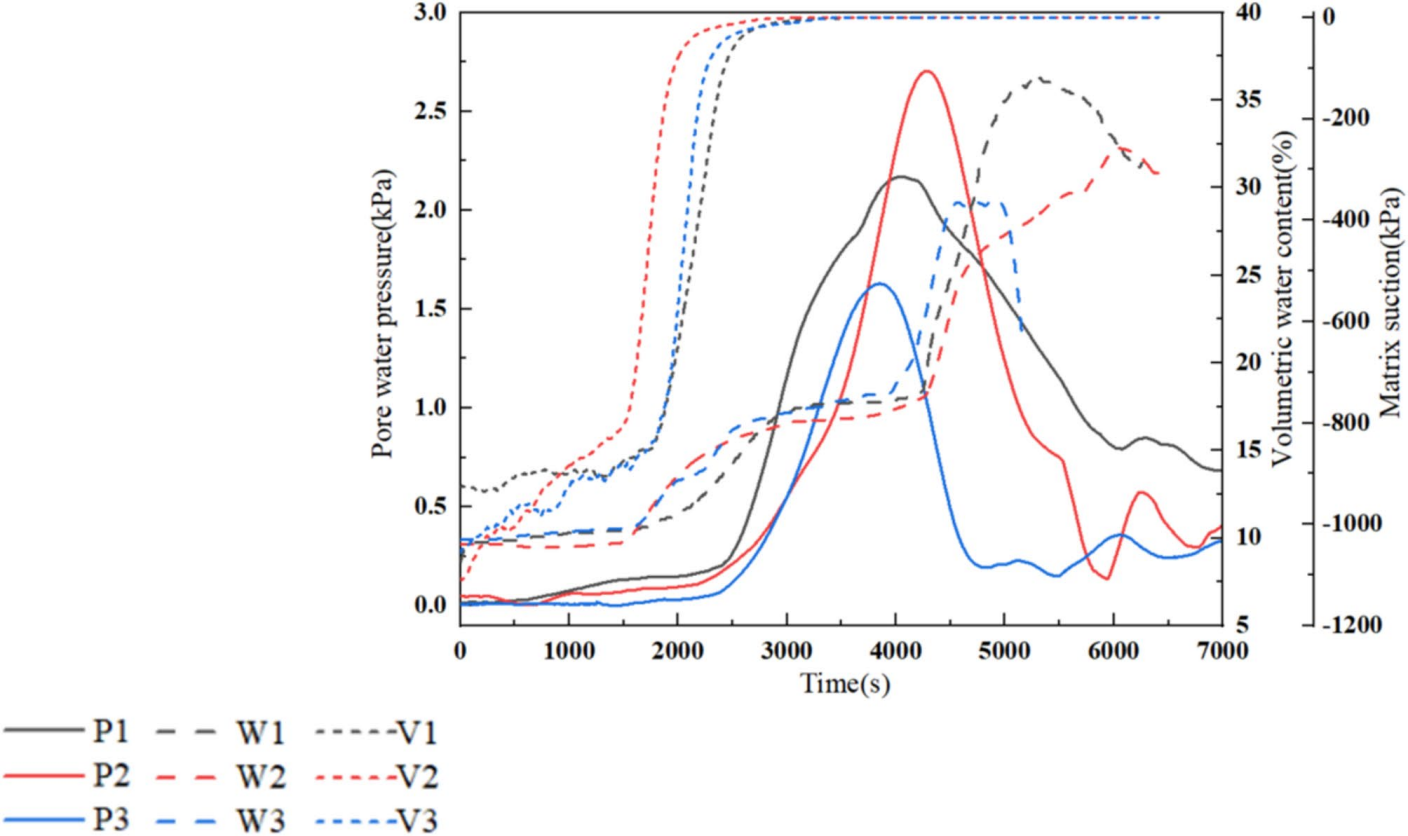
Hydrodynamic response curves of the arch-locked-segment landslide.

During the landslide, hydrodynamic parameters such as pore water pressure, moisture content, and matrix suction are closely related to slope stability. These parameters can reflect the physical indicators of moisture in the landslide soil, reveal the instability mechanisms of rainfall-induced landslides, and establish correlations between hydrodynamic parameters and landslide stability. Figure 6 shows the response curves for hydrodynamic parameters of the arch-locked-segment landslide. At the beginning of rainfall, rainwater is mainly in the form of slope runoff, and the pore water pressure, moisture content, and matric suction within the slope are changing slowly. As the infiltration intensity increases, the hydrodynamic parameters within the slope respond rapidly and exhibit certain variation patterns.

In the early rainfall stage, pore water pressure increases slowly due to the influence of gases within the pores. As rainfall continues, the slope transitions from an unsaturated to a saturated state, which increases the permeability coefficient and the gradual formation of stable seepage pathways, resulting in a rapid increase in pore water pressure. The rise in pore water pressure causes a decrease in effective stress and shear strength within the slope, accompanied by rapid pore water release. The volumetric water content primarily affects the permeability coefficient of unsaturated soil, influencing rainfall infiltration capacity. During the early stage of rainfall, most rainwater flows to the slope toe through the slope surface, resulting in low infiltration capacity and a slow increase in volumetric water content, and the duration of this stage is relatively short. As the rainfall continues, the infiltration capacity of the landslide increases, leading to a rise in volumetric water content. Subsequently, rainfall mainly infiltrates forward from the rear edge of the slope, with the volumetric water content reaching a relatively stable state. Afterward, the volumetric water content starts rising again, signifying reduced landslide stability. Matrix suction reflects the water absorption capacity of soil particles and is a crucial indicator for distinguishing unsaturated soils from saturated soils. As rainfall infiltrates, the matrix suction within the slope gradually increases from around − 1,300 kPa to 0 kPa. The dissipation process of matrix suction may lead to a reduction in the shear strength of unsaturated soil. In other words, as the moisture content of the landslide increases, the matrix suction dissipates rapidly, followed by the case that pore water pressure peaks within a short period. The pore water pressure decreases quickly as the landslide experiences instability while the moisture content rises.

### Analysis of the tilting deformation curves

**Fig. 7:**
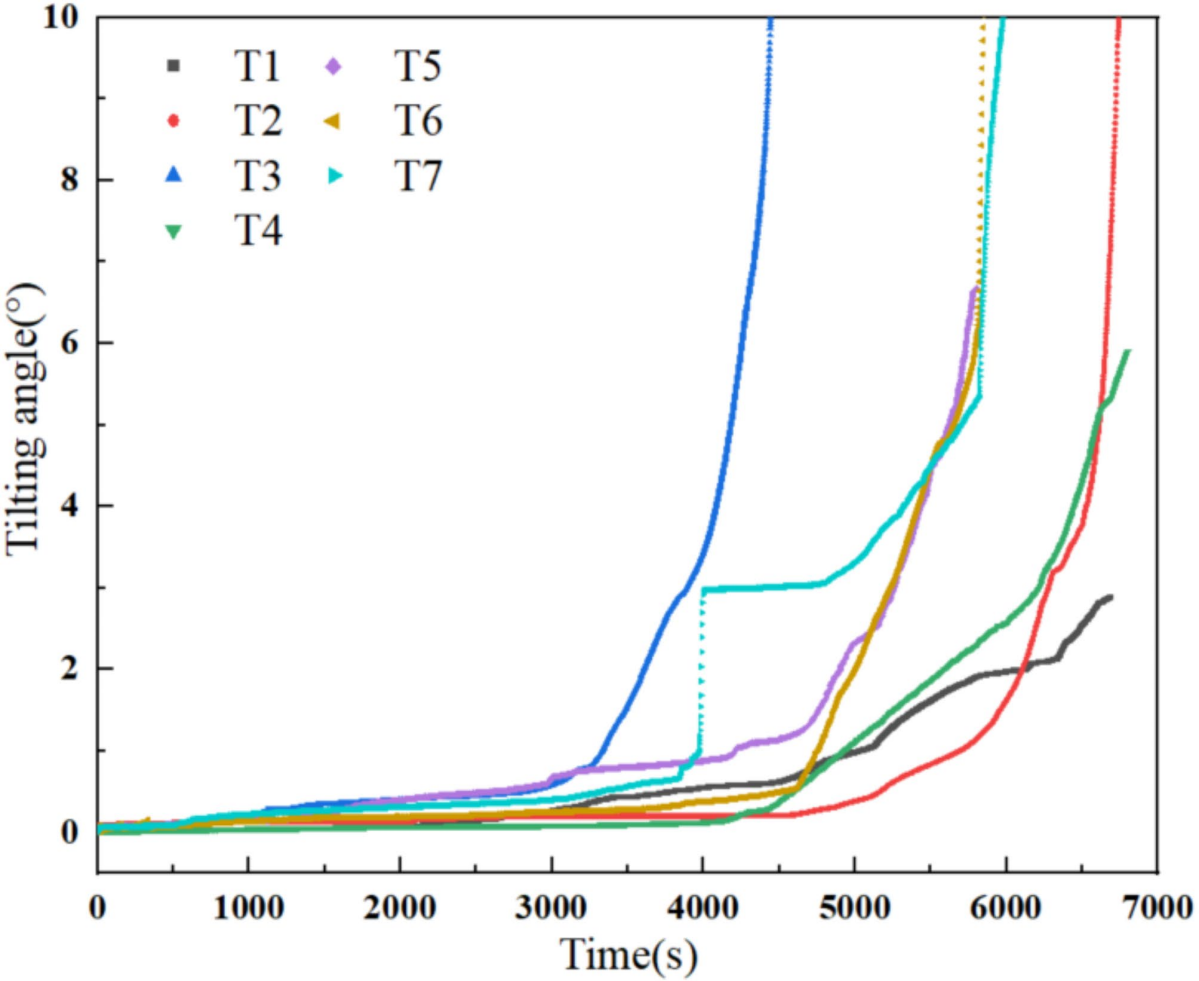
Tilting deformation curves of the arch-locked-segment landslide.

The tilting deformation curves (Fig. 7) for the rainfall-induced arch-locked-segment landslide indicate that in the early stage of rainfall, the landslide is in an initial creep stage, and its tilting deformation curves remain relatively stable. As rainfall continues, the landslide enters a constant-speed creep stage, slowly increasing the tilting deformation curves. Subsequently, the landslide enters the accelerated creep stage characterized by a significant increase in the rate of tilting deformation. Specifically, the surface tilt sensor T3 first enters the accelerated creep stage with rainfall and fails at around 4,400 s when it reaches the 90° tail. As rainfall continues, the tilting deformation curve at T7 for the locked segment suddenly increases at around 3,900 s, and the corresponding angle rises sharply to 3°, indicating fracture in the locked segment on one side. After this point, the tilting angle of the locked segment remains almost unchanged. Around 4,700 s after rainfall, the tilting deformation curve at T7 for the locked segment again enters an accelerated creep stage. Meanwhile, the tilting deformation curve at T6 for the locked segment on the other side also enters the accelerated creep stage, signifying that the locked segment of the landslide begins to collapse rapidly, leading to overall instability and sliding of the landslide. Also, deep tilt sensors T4 and T5, as well as tilt sensors T1 and T2 above the slope surface, enter the accelerated creep stage. The tilting deformation curves at T6 and T7 for the locked segment reach the 90° tail state at around 5,800 s after rainfall, indicating that the locked segment had completely lost its support, leading to instability and sliding of the landslide.

**Fig. 8:**
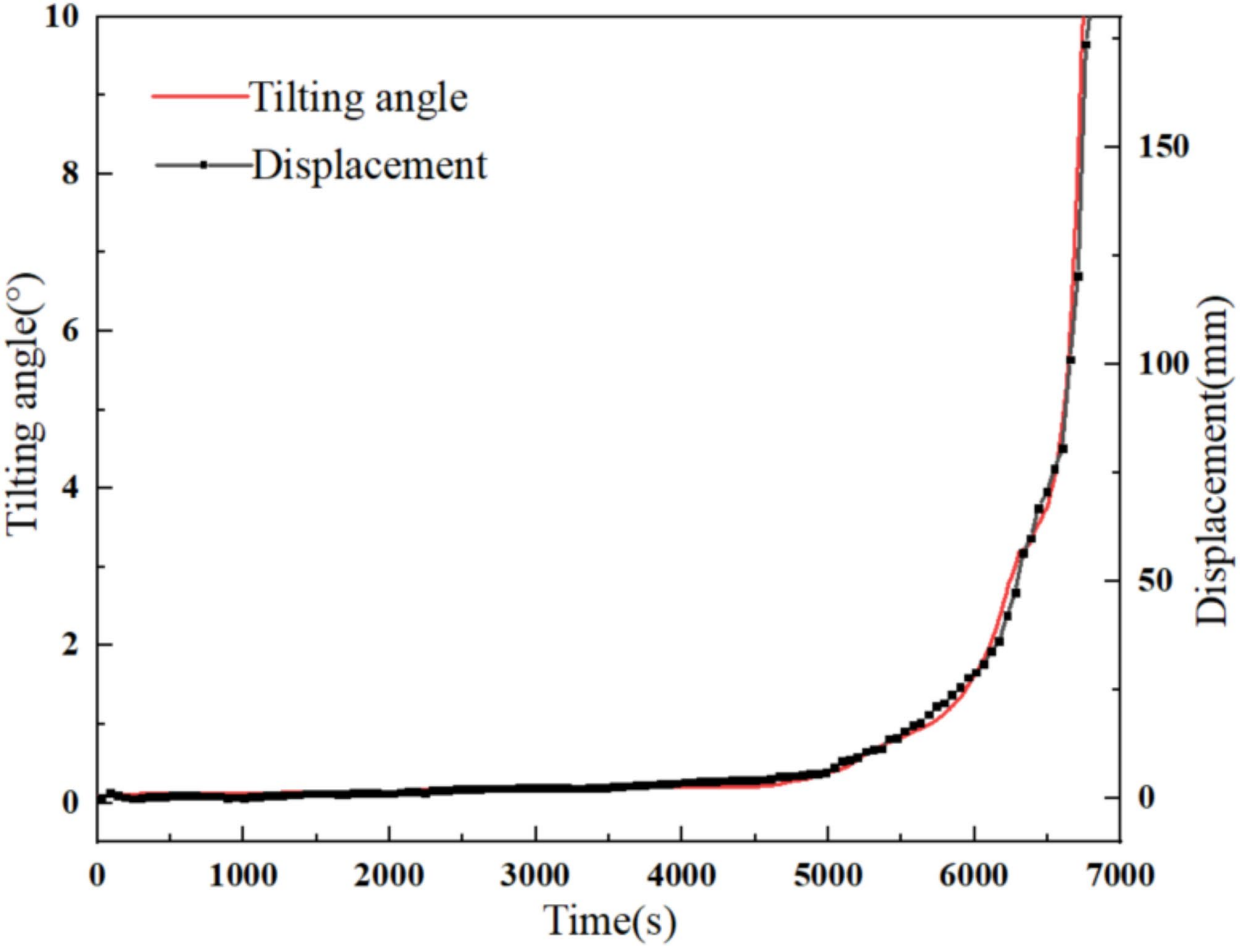
Variations of displacement and tilting angle at T2.

**Fig. 9:**
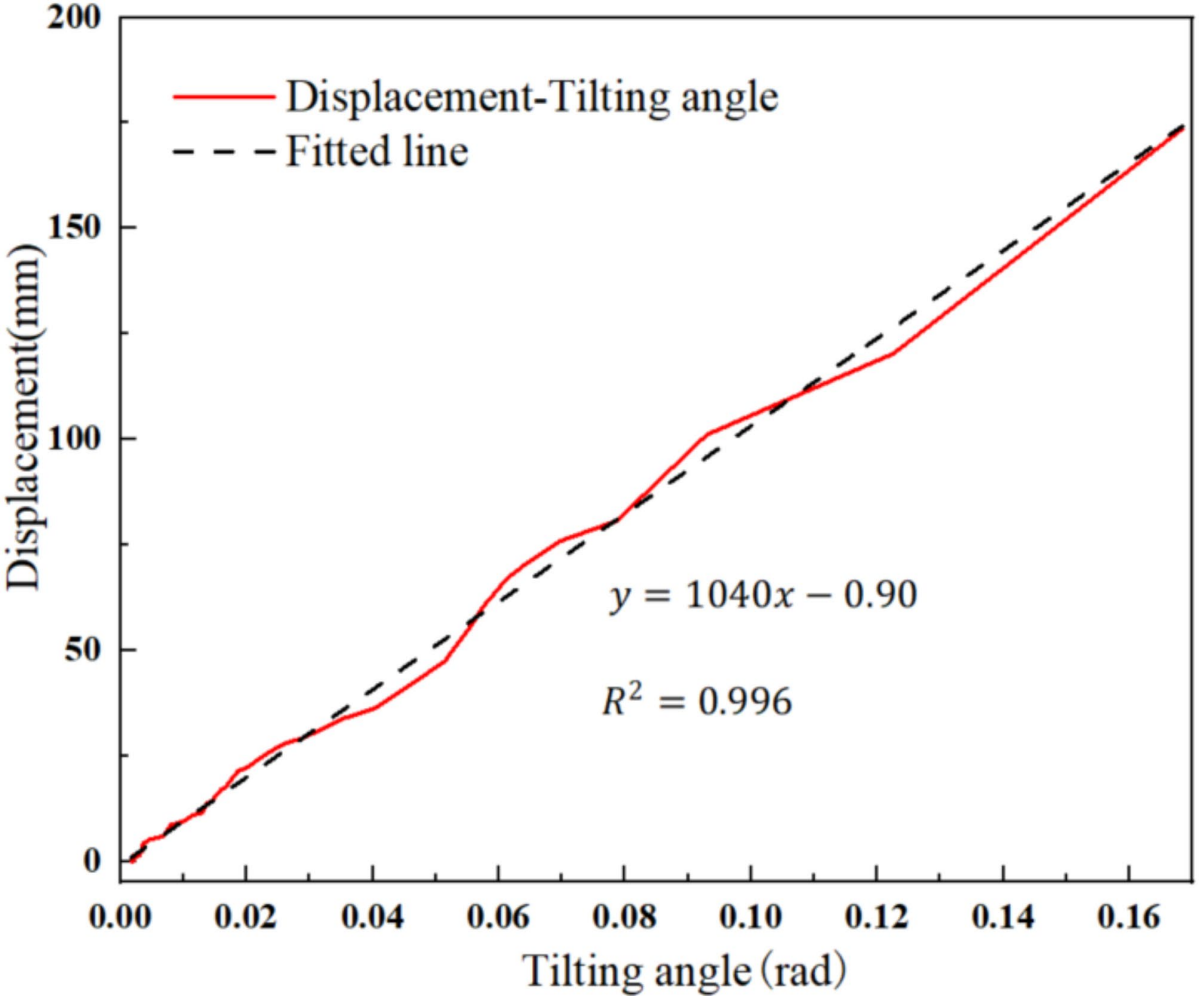
Displacement versus tilting angle curves at T2.

The tilting angle curves measured in degrees (°) were converted to curves measured in radians (rad), and the curves before and after conversion were found to be consistent. During the experiment, a DIC 3D full-field strain measurement system was used to analyze the changes in the displacement from the tilt sensors and investigate the relationship between tilting angle and displacement during landslide evolution. In the case of tilt sensor T2, as illustrated in Fig. 8, the converted tilting angle versus time and displacement versus time curves reveal consistent variations and exhibit the typical three-stage creep characteristics. When the tilting angle reaches its maximum, the corresponding displacement reaches its maximum. In order to further clarify the relationship between landslide displacement and tilting angle, an analysis was conducted using change in tilting angle as the *x*-axis and displacement variation as the *y*-axis. The corresponding displacement-tilting angle curves for the arch-locked-segment landslide exhibit a clear linear relationship. Fitting these curves yields corresponding fit lines (Fig. 9), corroborating the effectiveness of tilt sensors in landslide monitoring.

### Failure characteristics of the locked segment

**Fig. 10:**
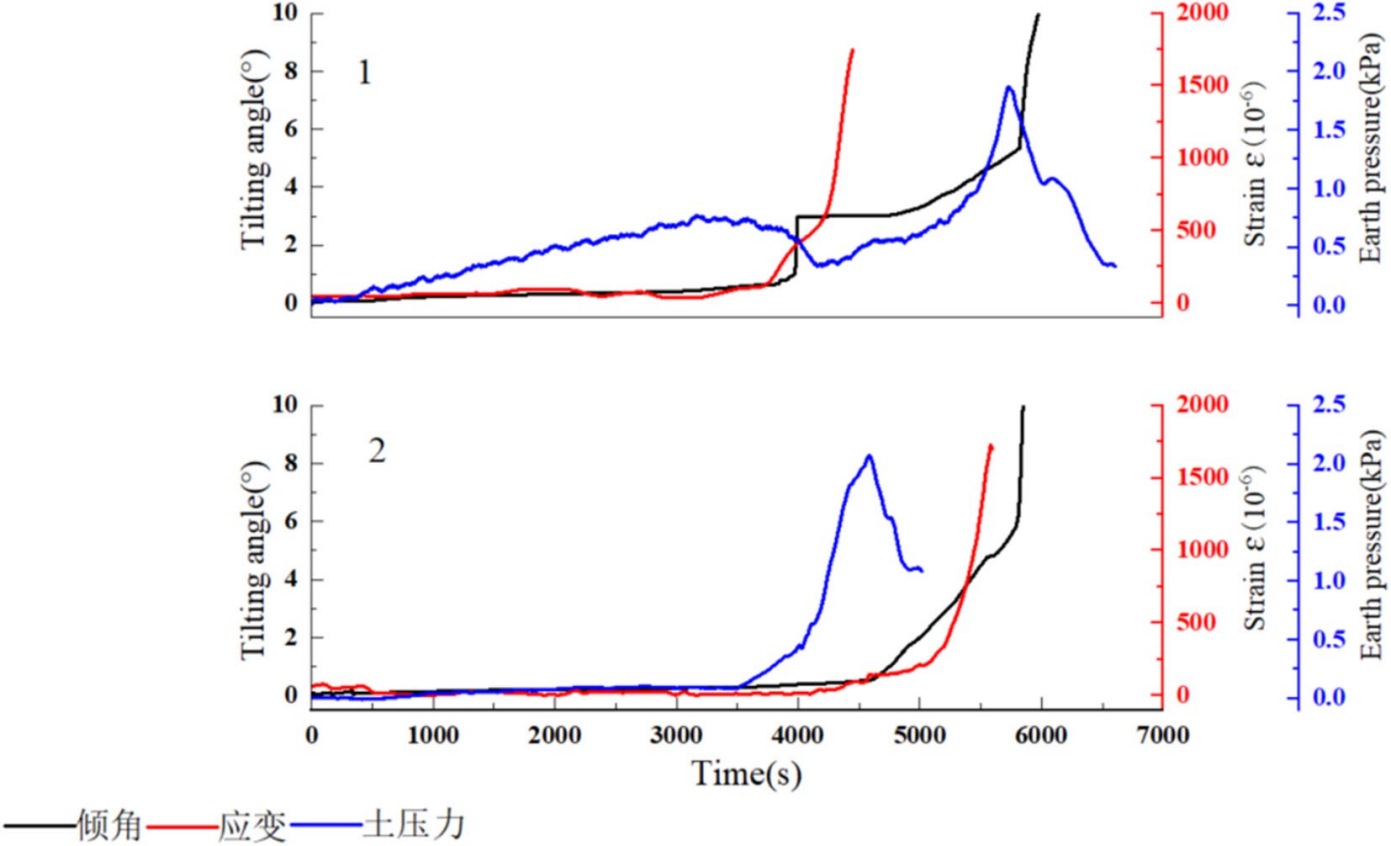
Characteristics of the locked segment of the arch locked-segment landslide.

The locked segment has a crucial role in landslide evolution. Based on the failure characteristics of the locked segment during the evolution of the arch locked-segment landslide (Fig. 10), the variation in tilting angle, strain, and earth pressure for locked segments 1 and 2 were analyzed to reveal the evolution mechanism of the tilting deformation. With rainfall infiltration, the earth pressure on locked segment 1 initially increases slowly. At around 3,200 s after rainfall, the earth pressure rises to 0.7 kPa and then gradually decreases. At approximately 3,700 s, the strain value for locked segment 1 increases sharply, and 200 s later, the tilting angle rises sharply to 2.8°, indicating that a fracture has occurred in the locked segment. After the fracture, the locked segment still provides some support. As the rear edge of the slope slides downward, the earth pressure on the locked segment continues to rise, and its tilting angle also increases slowly. At approximately 5,700 s, the earth pressure on the locked segment rapidly increases to a peak of about 1.85 kPa and then decreases rapidly. The decreased earth pressure indicates that the locked segment rapidly loses its supporting function. At 5,800 s of rainfall, as earth pressure decreases, the tilting angle of the locked segment suddenly rises to 10°. An analysis of locked segment 2 on the opposite side of landslide M1-2 reveals significant differences in the deformation curves between these two segments, which suggests that the locked segments on both sides of the arch locked-segment landslide M1-2 do not fracture simultaneously. Also, the differences in the landslide and locked segments lead to variations in the evolution processes of the locked segments. Within the 3,500 s of rainfall, the tilting angle, strain, and earth pressure curves for the locked segment exhibit minor fluctuations, indicating that the locked segment on this side is stable. After 3,500 s of rainfall, earth pressure in the locked segment gradually increases and peaks at around 4,600 s. Then, it drops rapidly, accompanied by the strain curves and tilting angle curves entering the rapidly increasing stage. The strain gauges are located at the fracture point of the locked segment, while the tilt sensors are positioned on the edge side of the locked segment. Therefore, the strain curve reaches its range and fails before the tilting angle curve, with the tilting angle curve lagging behind the strain curve by 100 s.

### Prediction of landslide instability time

Based on research on the displacement tangent angle ^40^, the tilting deformation curve of the landslide using the Angle-tangent angle was further analyzed. The tilting deformation curve was first subjected to a unified coordinate transformation using Eq. (1). Based on tangent angles of 80° and 85°, the accelerated creep stage of the curve was further divided into initial acceleration, medium acceleration, and high acceleration stages. On this basis, the tangent angle criterion for the landslide angle was proposed.

1 $$\:T\left(i\right)=\frac{\theta\:\left(i\right)}{v}$$

where, $\:T\left(i\right)$ is the value of the longitudinal coordinate with the same magnitude as time after the coordinate transformation, $\:\theta\:\left(i\right)$ is the cumulative angle, and $\:v$ is the tilting rate in the constant velocity creep stage.

**Fig. 11:**
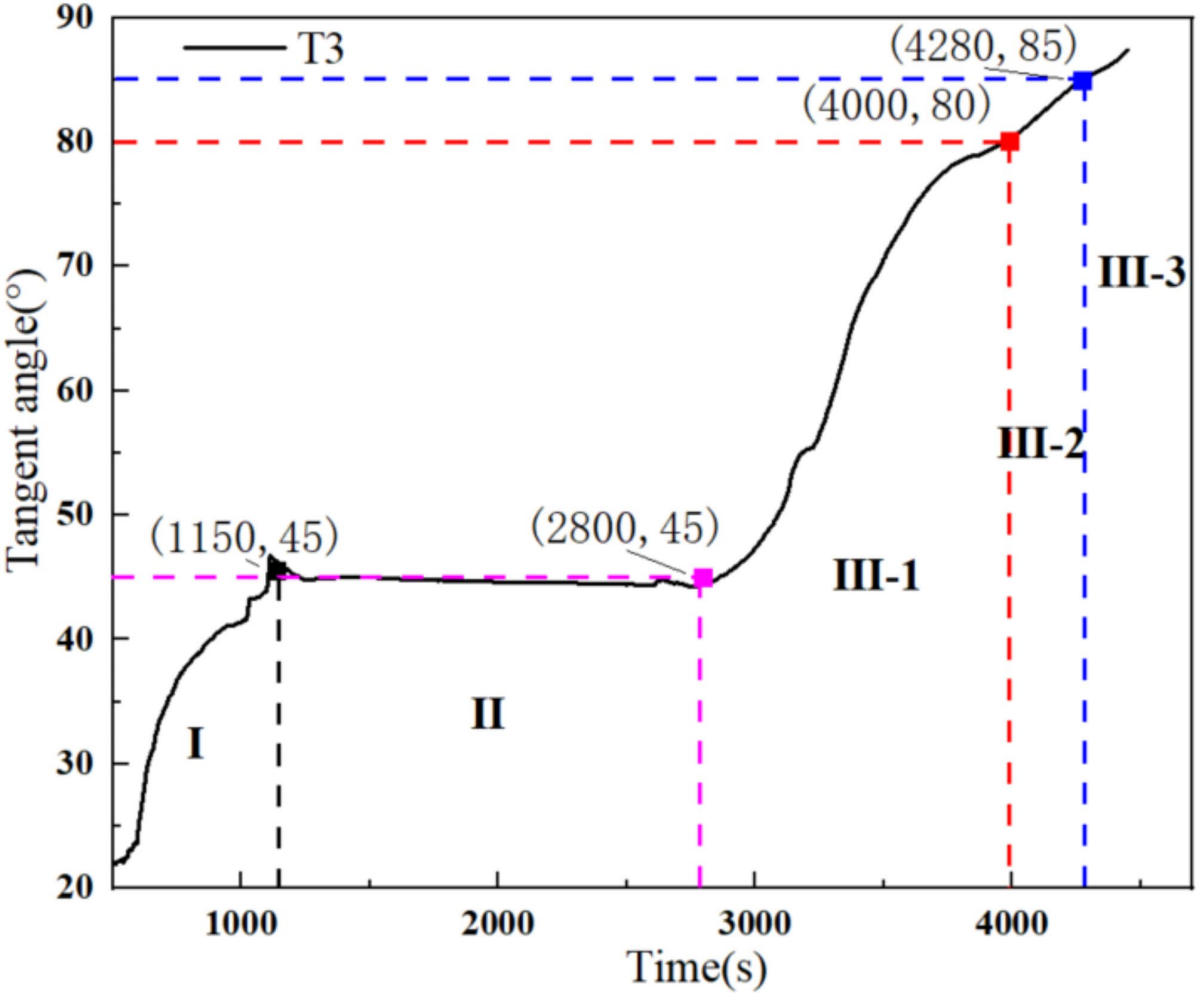
Different stages of tangent angle-time curve.

**Fig. 12:**
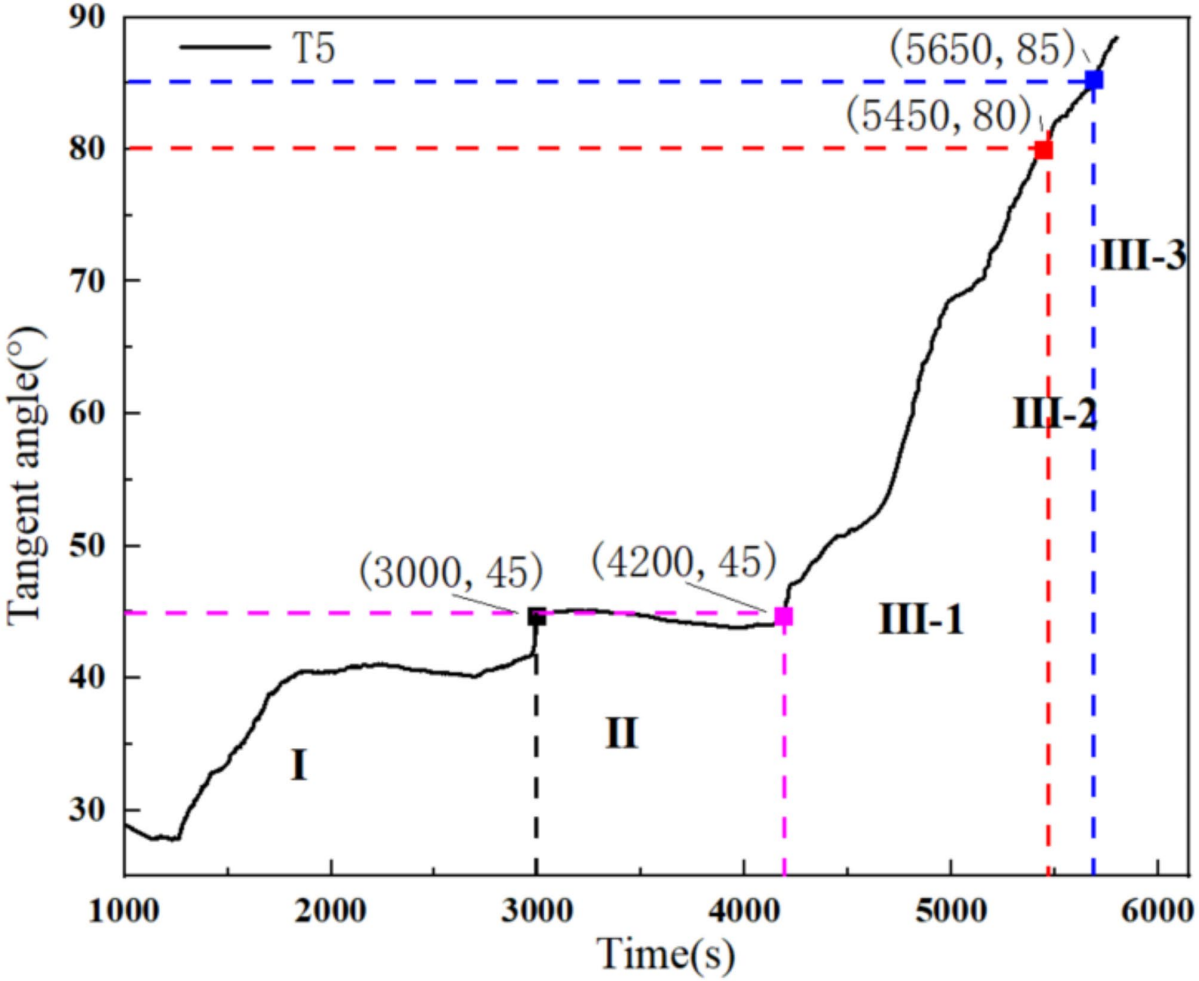
Different stages of tilting deformation curve.

Considering the tilting deformation curve at T5 from the arch locked-segment landslide as an example, the corresponding rainfall times are firstly determined based on the tangent angle-time curve at 45°, 80°, and 85° (Fig. 11). Subsequently, the tilting angles at various monitoring points of the landslide were determined based on these rainfall times (Fig. 12). The tilting deformation curve at T5 is divided into the initial creep stage (~ 3,000 s), constant velocity creep stage (3,000 s – 4,200 s), initial acceleration creep stage (4,200 s – 5,450 s), medium acceleration creep stage (5,450 s – 5,650 s), and high acceleration creep stage (5,650 s~). The corresponding tilting angles for initial, medium, and high acceleration creep stages are 0.97°, 4.10°, and 5.17°, respectively. This provides new perspectives and methods for studying landslide instability failure, which is crucial for improved early warning capability for landslide hazards and reducing hazard-related loss.

Further, a method for predicting landslide instability time was proposed based on the tangent angle method for the arch-locked-segment landslide angle ^32^. The tilting rate reciprocal method was utilized to investigate the accelerated creep stage of the tilting deformation curve and establish the predicting criteria. The relation for calculating the tilting rate reciprocal is expressed as Eq. (2).

2 $$\:\frac{dt}{\left|d\theta\:\right|}=\frac{{t}_{ij}-{t}_{ij-1}}{d\theta\:}=\frac{-t}{B}+\frac{{t}_{f}}{B}$$

where, $\:{\text{t}}_{\text{i}\text{j}}$ and $\:{\text{t}}_{\text{i}\text{j}-1}$ denote time; $\:d\theta\:$ is the increment of the tilting angle from $\:{\text{t}}_{\text{i}\text{j}-1}$ to $\:{\text{t}}_{\text{i}\text{j}}$; $\:{\left(\frac{\left|d\theta\:\right|}{dt}\right)}_{ij}$ is the tilting rate reciprocal at $\:{\text{t}}_{\text{i}\text{j}}$; and $\:B$ is the angle coefficient derived from this linear relationship; $\:{t}_{f}$ is the instability failure time of the landslide by assuming that the reciprocal of this tilting rate is 0 (s/°) (i.e., $\:\frac{dt}{\left|d\theta\:\right|}=0$).

**Fig. 13:**
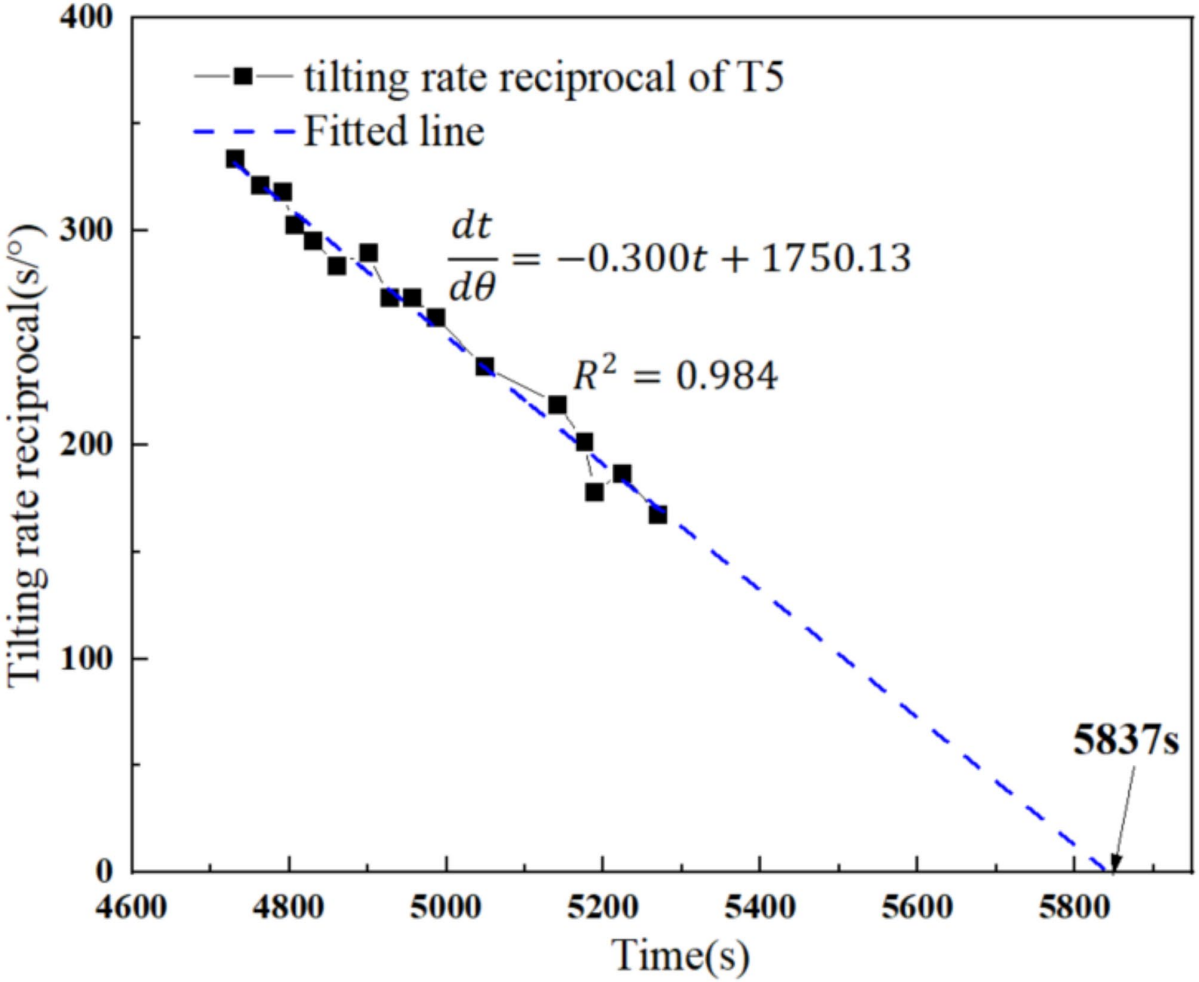
Tilting rate reciprocal versus time curves at T5.

An analysis was conducted with tilt sensor T5 from the arch-locked-segment landslide, as shown in Fig. 13. The data collection was unstable due to external rainfall conditions and monitoring equipment. Therefore, noise reduction was conducted during data processing, and the angle values from the initial acceleration creep stage were selected to establish a data series at an interval of 0.5°. Using Eq. 2, the initial acceleration creep stage of the tilting deformation curve at T5 was transformed into a relationship between the tilting rate reciprocal and the time, and a linear fit was made. The fitted line for the tilting rate reciprocal in this stage was set to 0 s/°, corresponding to the 5,837 s, i.e., the predicted failure time for the landslide area at T5. According to the tilting deformation curve at T5, the actual failure time for this area is 5,770 s, indicating that the predicted time aligns closely with the actual time. Therefore, an initial judgment can be made regarding its stability, enabling the implementation of appropriate warning and predicting measures by assessing the tilting angle of the landslide.

## Case study

**Fig. 14:**
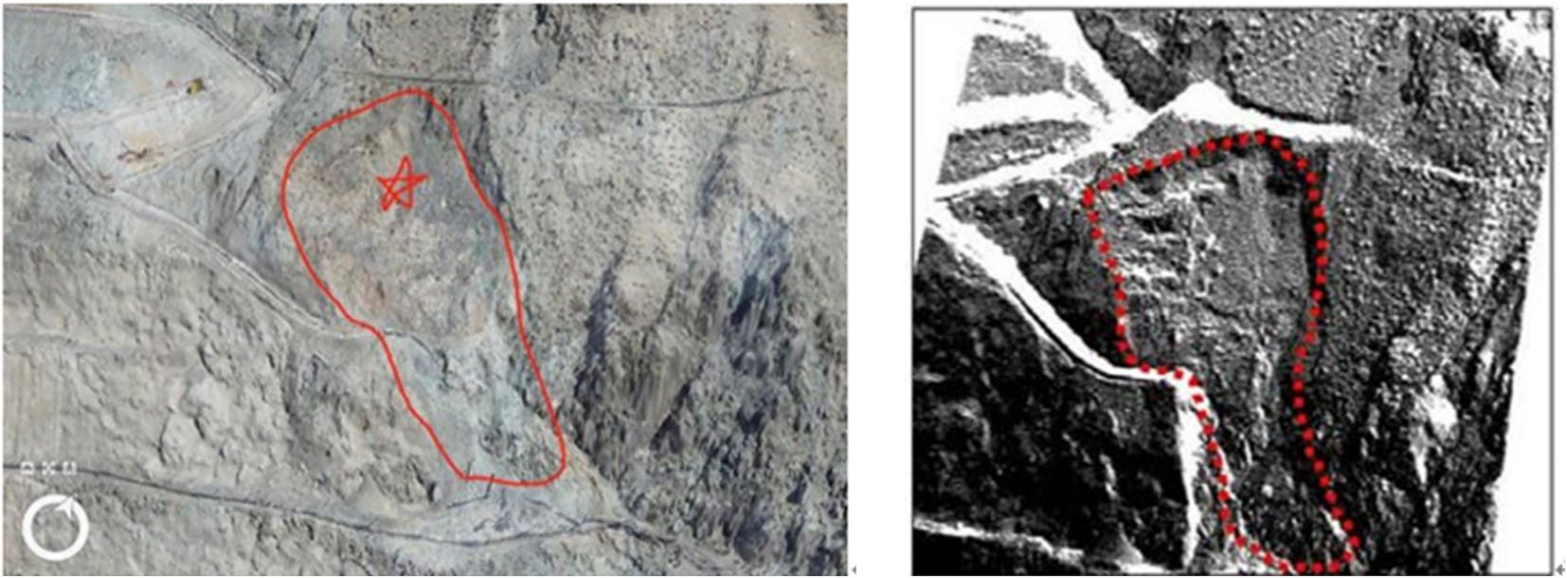
HB2 landslide in the unstable area.

The monitoring data from physical model tests of the rainfall-induced arch-locked-segment landslide was analyzed to understand the evolution of tilting deformation curves. The instability failure mechanism of this type of landslide was revealed, and landslide instability criteria were proposed based on the tilting deformation curves. In order to further validate the effectiveness of tilting deformation monitoring, a case study was conducted on the HB2 landslide at the Huangzangsi Hydraulic Hub site (Fig. 14).

**Fig. 15:**
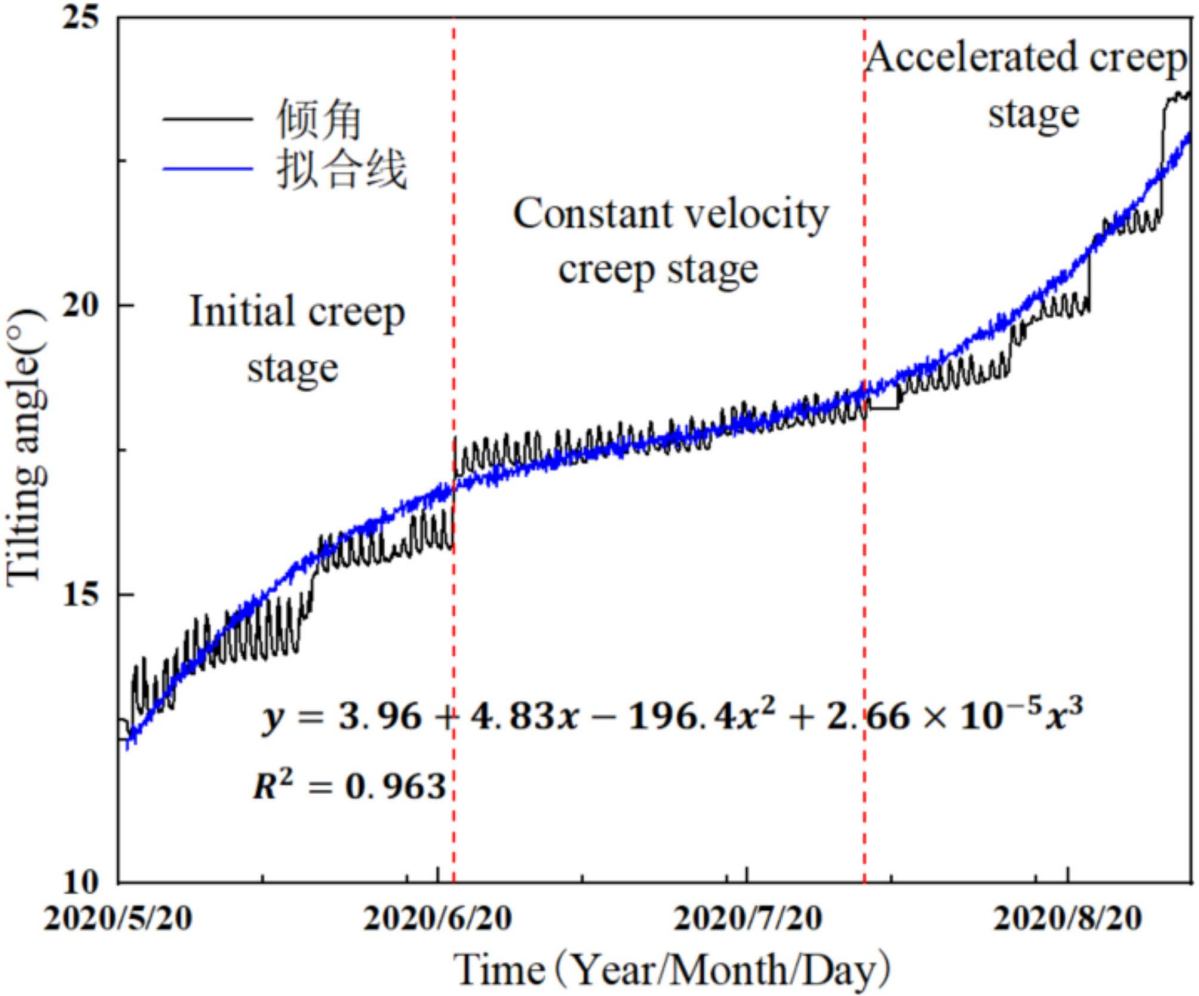
Tilting deformation monitoring curve for landslide HB2.

During the construction of the Huangzangsi Hydraulic Hub, excavation and rainfall led to the sliding failure of the HB2 landslide by the tilting deformation curve, as shown in Fig. 15. The tilting deformation curve of the HB2 landslide was further divided into initial creep stage, constant velocity creep stage, and accelerated creep stage based on the creep characteristics. The monitoring period from May 20, 2020, to June 21, 2020, was classified as the initial creep stage, during which the tilting angle gradually increases, and the tilting rate changes slowly with the decreasing trend. The monitoring period, from June 22, 2020, to July 31, 2020, was classified as the constant velocity creep stage, during which the tilting angle changes almost linearly, indicating a continuous tilting rate. The monitoring period from August 1, 2020, to September 1, 2020, was identified as the accelerated creep stage. During this stage, the tilting angle keeps rising, and the tilting rate gradually increases, indicating that the HB2 landslide area enters an accelerated failure stage and ultimately experiences a complete collapse.

**Fig. 16:**
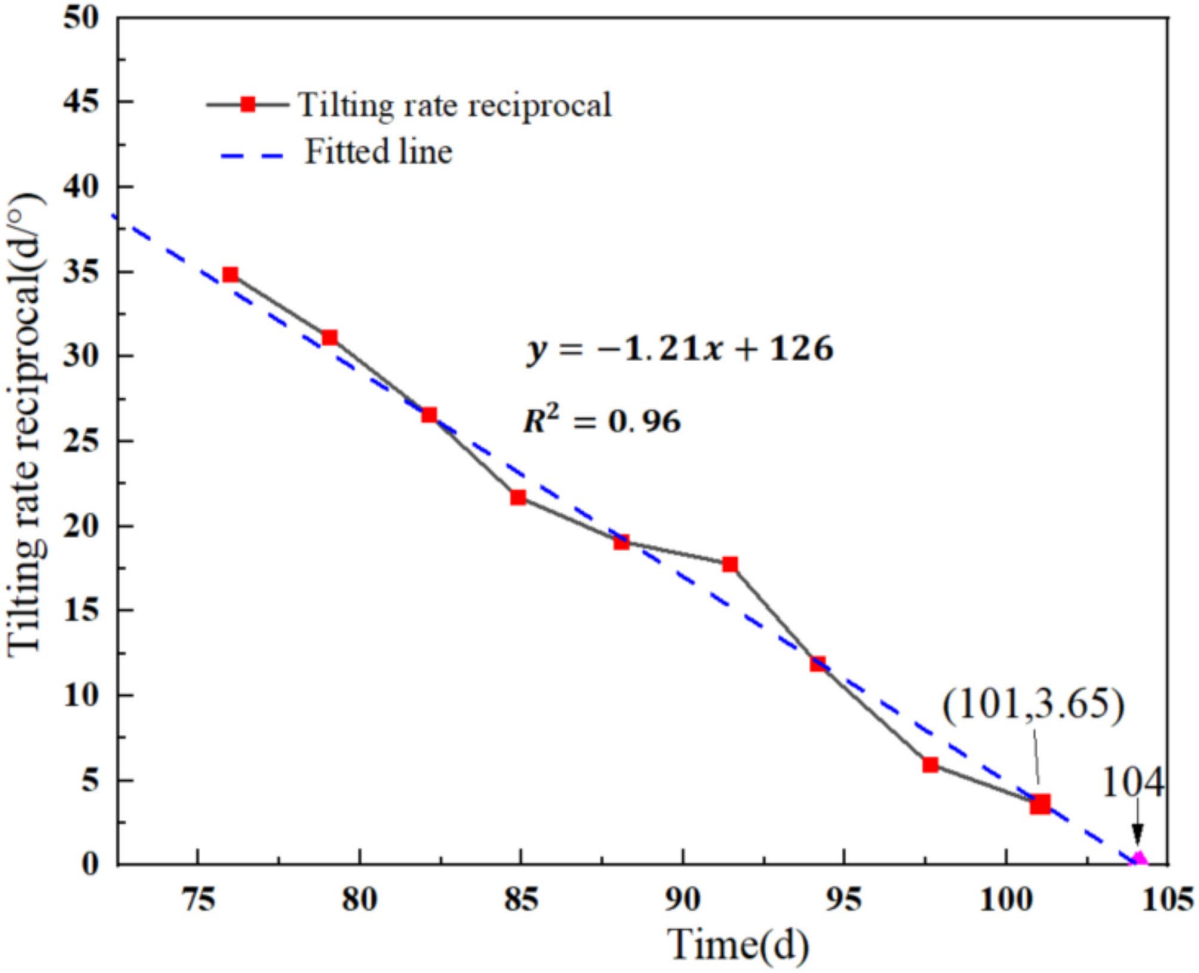
Variation of tilting rate reciprocal for landslide HB2.

Based on the aforementioned physical model test of the arch locked-segment landslide, the tilting rate reciprocal method is applied to predict the instability time of the tilting deformation curve for the HB2 landslide. This yields a curve showing the relationship between the tilting rate reciprocal and the time (Fig. 16). The time calculated when the tilting rate reciprocal equals zero (d/°) is considered the instability failure time of the landslide. Thus, the failure time of the HB2 landslide is predicted to be 104 days, while the actual failure occurred at 101 days. Comparative analysis indicates that the predicted instability failure time of the landslide based on the tilting rate reciprocal method closely aligns with the actual time. Therefore, it is reliable to predict the failure instability time of the landslide based on tilting deformation curves.

## Conclusions

In this study, physical model testing and field monitoring were conducted for the arch-locked-segment landslide to investigate its tilting deformation and instability mechanism under rainfall conditions. Hydrodynamic response, slope morphology, displacement nephograms, and failure characteristics of the locked segment corroborated the effectiveness of tilt sensors in landslide monitoring and prediction. The evolution characteristics of tilting deformation and the instability mechanism were analyzed, and methods for predicting landslide instability based on tilting deformation curves were proposed. The main conclusions drawn from the results obtained are as follows.

(1) The tilting deformation evolution curve of the arch-locked-segment landslide under rainfall conditions exhibits typical three-stage creep characteristics. The failure mode exhibits a front-edge traction and rear-edge sliding failure. The arch-locked segment plays a significant role in controlling landslide stability, and an arch-like structure may form in this segment.

(2) The hydrodynamic parameter response curves of the arch-locked-segment landslide exhibit consistent changes under rainfall conditions, which include the stages of a gradual increase, rapid increase, and rapid decrease in pore water pressure, i.e., an increase, stabilization, and subsequent increase in the moisture content, with a gradual decline followed by a rapid drop to zero in matric suction. These changes are related to the adsorption and release of moisture in the soil, and the interactions of these factors collectively influence the landslide stability.

(3) The failure characteristics of the locked segment of the arch locked-segment landslide were analyzed. The curves of earth pressure, strain, and tilting deformation in the locked segment exhibit synchronous changes during the deformation and failure process, validating the accuracy and effectiveness of tilt sensors in landslide monitoring. Additionally, the tilting deformation curve in the locked segment can be used to assess its failure state and predict the landslide evolution. Similarly, relevant instability criteria for landslide evolution can be proposed based on any tilting deformation monitoring curve for any landslide.

(4) Based on the results of physical model testing, methods for classifying the landslide stages based on the tangent angle method and for predicting the landslide instability time based on the tilting rate reciprocal method were proposed, effectively validating the case study of the HB2 landslide at the Huangzangsi site. The instability failure time is determined through fitting and calculation by examining the linear relationship between the tilting rate reciprocal and the time throughout the landslide’s evolution process. A new method for predicting the instability of the arch-locked-segment landslide was thus proposed, providing a theoretical basis for understanding catastrophic mechanisms and instability prediction of landslides.

## Data Availability

All data are available in the paper or from the corresponding author on request.
